# Development of Microfluidic Systems Enabling High-Throughput Single-Cell Protein Characterization

**DOI:** 10.3390/s16020232

**Published:** 2016-02-16

**Authors:** Beiyuan Fan, Xiufeng Li, Deyong Chen, Hongshang Peng, Junbo Wang, Jian Chen

**Affiliations:** 1 State Key Laboratory of Transducer Technology, Institute of Electronics, Chinese Academy of Sciences, Beijing 100190, China; fanbeiyuan@ucas.ac.cn (B.F.); lixiufeng13@mails.ucas.ac.cn (X.L.); dychen@mail.ie.ac.cn (D.C.); 2 School of Science, Minzu University of China, Beijing 100081, China; hshpeng@muc.edu.cn

**Keywords:** microfluidics, single-cell analysis, protein characterization, high-throughput

## Abstract

This article reviews recent developments in microfluidic systems enabling high-throughput characterization of single-cell proteins. Four key perspectives of microfluidic platforms are included in this review: (1) microfluidic fluorescent flow cytometry; (2) droplet based microfluidic flow cytometry; (3) large-array micro wells (microengraving); and (4) large-array micro chambers (barcode microchips). We examine the advantages and limitations of each technique and discuss future research opportunities by focusing on three key performance parameters (absolute quantification, sensitivity, and throughput).

## 1. Introduction

Quantification of single-cell proteomics (e.g., functional proteins such as cytokines, and structural proteins such as actin filaments) provides key insights in the field of cellular heterogeneity (e.g., immune response variations and tumor heterogeneity) [1,2,3,4,5,6]. Fluorescent flow cytometry is the dominant workhorse in the field of single-cell protein quantification, featured with high throughput and high levels of multiplexing [7,8,9,10]. However, this technique is a semi-quantitative approach, reporting the intensity of fluorescence rather than the copy number of target proteins for individual cells. Even within the incorporation of calibration beads, the capability of absolute quantification is still questionable [11,12,13,14,15].

Microfluidics is the science and technology on the processing and manipulation of small amounts of fluids (10^−9^ to 10^−18^ L) in channels with dimensions of tens of micrometers [16,17,18]. The micrometer dimension matches well with the size of typical biological cells, making microfluidics an ideal platform for cell studies [19,20,21,22,23], including the characterization of biochemical (e.g., gene [24,25] and protein [6,26,27,28,29,30,31]) and/or biophysical properties (mechanical [32,33,34,35,36] and electrical [33,37,38,39]) of cells at the single-cell level [40,41,42,43,44]. More specifically, microfluidic systems can effectively confine individual cells in droplets and/or micro wells, enabling the absolute quantification of secreted proteins or cytosolic proteins of single cells, which cannot be easily quantified by conventional techniques such as fluorescent flow cytometry [26,27,28,29].

Within the last ten years, we have witnessed huge developments in microfluidic platforms enabling single-cell protein expressions (see Table 1), which can be classified into four major types as follows: (1) microfluidic fluorescent flow cytometry; (2) droplet based microfluidic flow cytometry; (3) large-array micro wells (microengraving); and (4) large-array micro chambers (barcode microchips). In this mini-review, we examine the advantages and limitations of each technique and discuss future research opportunities by focusing on three key performance parameters (absolute quantification, sensitivity, and throughput).

**Table 1:** Key developments of microfluidic systems enabling high-throughput single-cell protein characterization.

Techniques	Key Achievements	References
Microfluidic Fluorescent Flow Cytometry	Characterization of small numbers of cells, ranging from 20,000 to 625 per sample	[45,46,47]
Droplet Based Microfluidic Flow Cytometry	Detection of yellow fluorescent protein mutant “Venus” of single *E. coli* encapsulated in microdroplets	[48]
Droplet Based Microfluidic Flow Cytometry	Detection of the activities of enzyme alkaline phosphatase secreted by single *E. coli* encapsulated in microdroplets	[49]
Droplet Based Microfluidic Flow Cytometry	Detection of cytokine (IL-10) secretion of single CD4+ CD25+ regulatory T cells in microdroplets over time	[50]
Droplet Based Microfluidic Flow Cytometry	Detection of intracellular proteins of HRas-mCitrine, expressed within single HEK-293 cells and actin-EGFP expressed within single MCF-7 cells encapsulated in microdroplets	[51]
Droplet Based Microfluidic Flow Cytometry	Detection of cytokine (IL-2, IFN-γ, TNF-α) secretion of single, activated T-cells in microdroplets over time	[52]
Large-Array Micro Wells (Microengraving)	Detection of secreted cytokines (IL-6, IL-17, IFN-γ, IL-2, and TNF-α) of primary T cells at the secretion rate from 0.5 to 4 molecules/s	[53]
Large-Array Micro Wells (Microengraving)	Detection of secreted cytokines (IFN-γ and IL-17) of individual CD4+ T cells with peptide-loaded MHC class II pre-coated on the surface of micro wells for on-chip activation	[54]
Large-Array Micro Wells (Microengraving)	~200-fold improvement in the limits of detection of secreted cytokines using hybridization chain reactions	[55]
Large-Array Micro Wells (Microengraving)	Detection of serial, time-dependent secreted cytokines (IFN-γ, IL-2, TNF-α) of primary human T cells, revealing that cells predominantly release one cytokine at a time rather than actively secret multiple cytokines simultaneously	[56]
Large-Array Micro Wells (Microengraving)	Detection of secreted chemokines (ELR + CXC) from single colorectal tumor and stromal cells with polyfunctional heterogeneity located	[57]
Large-Array Micro Chambers (Barcoding Microchips)	Detection of 12 proteins including TNF-α, IFN-γ, IL-2, IL-1α, IL-1β, IL-6, IL-10, IL-12, granulocyte-macrophage colony-stimulating factor, CCL-2, TGF-β and PSA of macrophages and cytotoxic T lymphocytes	[58]
Large-Array Micro Chambers (Barcoding Microchips)	Detection of 11 proteins directly or potentially associated with PI3K signaling of three isogenic cell lines representing the cancer glioblastoma multiforme, at the basal level, under EGF stimulation, and under erlotinib inhibition plus EGF stimulation	[59]
Large-Array Micro Chambers (Barcoding Microchips)	Detection of secreted proteins (IL-8 and VEGF) of circulating tumor cells	[60]

## 2. Microfluidic Fluorescent Flow Cytometry

**Figure 1:**
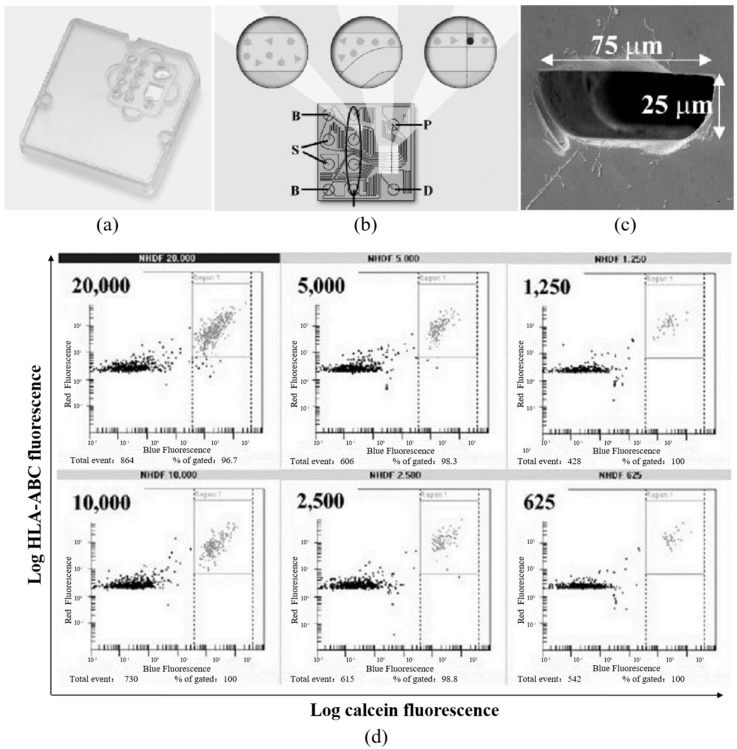
The commercially available microfluidic fluorescent flow cytometry enabling the quantification of single-cell protein expressions. (**a**) Schematic of the instrument for the detection of cellular fluorescence parameters; (**b**) chip layout of the microfluidic glass chip where each sample channel is joined by a buffer channel in close proximity to the detection area, focusing sample solutions to a portion of the microchannel in order to generate a single file cell stream; (**c**) the glass-based microfluidic channels with channel dimensions of 25 × 75 μm; and (**d**) low number of cells was loaded into the microfluidic flow cytometry with specific membrane proteins quantified. Reproduced with permission from [47].

In microfluidics, the fluorescent micro flow cytometry is the first approach proposed to quantify single-cell protein expressions, which is a miniaturized version of conventional flow cytometry [61,62,63,64]. As shown in Figure 1, single cells with surface (fluorescence labeled antibodies targeting surface proteins) or intracellular staining (fluorescence labeled antibodies targeting cytosolic proteins) are flushed into microfabricated flow channels. By integrating laser sources and fluorescent detection units, the fluorescent intensities of labeled single cells are obtained and the copy numbers of targeted proteins are quantified using the calibration curves obtained by calibration beads [46,47].

Compared to conventional flow cytometry, microfluidic flow cytometry is featured with a reduction in cellular samples and reagents. As shown in Figure 1d, the proposed system was used to analyze small numbers of cells, with the quantified number of cells decreased from 20,000 to 625. Thus, microfluidic flow cytometry is more suitable for protein quantification of small cell samples (e.g., primary cells or tumor cells obtained from biopsies). As to the capability of absolute quantification, both approaches can assay cell surface markers with absolute quantification by combining calibration beads. As to the measurement of intracellular proteins, due to the lack of effective calibration beads, flow cytometry-based approaches are still qualitative rather than quantitative, which needs further research efforts.

## 3. Droplet Based Microfluidic Flow Cytometry

**Figure 2:**
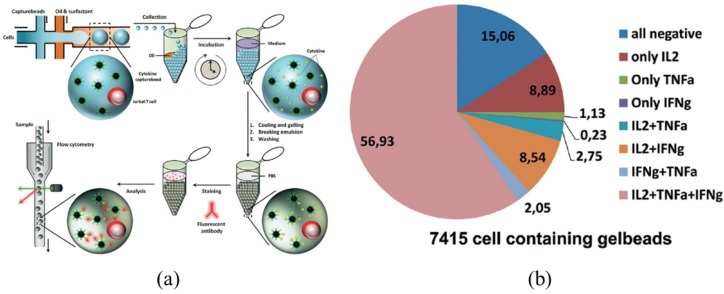
The droplet based microfluidic flow cytometry enabling the quantification of proteins released from single cells. (**a**) device schematic where single cells and functionalized cytokine-capture beads are encapsulated in monodisperse agarose droplets, which are then quantified by fluorescence flow cytometry; and (**b**) 7415 single cells are analyzed, revealing the presence of eight different cellular subpopulations where 85% of all individual cells secreted one or more cytokines. Reproduced with permission from [52].

Since microfluidic fluorescent flow cytometry cannot estimate the secreted proteins from single cells, droplet-based microfluidic flow cytometry [65,66,67] was modified to encapsulate single cells, enabling the quantification of specific proteins secreted by single cells [48,49,50,51,52,68,69,70,71,72,73,74,75]. As shown in Figure 2, single cells and functionalized capture beads are encapsulated in agarose-gel droplets, where beads function to bind cytokines secreted by single cells. Then, droplets are gelled and washed to break the emulsion, followed by incubation with fluorescently-labeled detection antibodies targeting cytokines bound on the beads. Subsequently, the signal intensities in the beads are quantified by flow cytometry. Based on this approach, 7415 single cells were analyzed, revealing that (1) there was a presence of eight different cellular subpopulations; and (2) 85% of all individual cells secreted one or more cytokines [52].

Although powerful, there are still several concerns for this technique, which need to be carefully addressed. (1) The cell loading efficiency in individual droplets needs to be further quantified and optimized. High concentrations of suspension cells before the emulsification process lead to loading of more than one cell in each droplet, while low concentrations of cell suspensions result in high percentages of empty droplets without single cells. Variations in fluorescent intensities resulted from uncontrolled cell numbers per droplet may compromise the quantitative results; (2) The local microenvironments of single cells trapped in individual droplets (e.g., nutrient levels and gas permeability) may be significantly different from *in vivo* situations and, thus, there is a doubt on the normal metabolism status of individual cells trapped in droplets. If these individual cells are confronted with challenging environments, the secreted protein numbers cannot indicate the normal status of these individual cells; and (3) the use of cytokine-capture beads also brings more issues. One consideration is the bead loading evenness among individual droplets, which may lead to variations in protein quantification. The second point is the calibration issue. Due to the use of cytokine-capture beads, there is an uneven distribution of fluorescence among individual droplets (intensity peaks around individual beads), which poses further obstacles in calibration.

**Figure 3:**
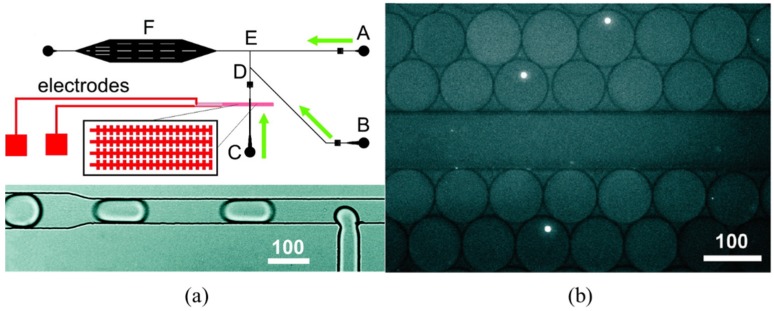
Schematic (**a**) and experiment results (**b**) of droplet-based microfluidic flow cytometry enabling the quantification of intracellular proteins of single cells including key steps of cellular lysis, encapsulation of cell lysate, and antibody-functionalized beads to form droplets, on-chip storage, and fluorescence reading. Reproduced with permission from [51].

In addition to the quantification of secreted proteins of single cells, droplet-based microfluidic systems have also been used to quantify intracellular proteins of single cells. As shown in Figure 3, cells are introduced into the device in suspension and are electrically lysed *in situ*. The cell lysate is subsequently encapsulated together with antibody-functionalized beads into droplets, which are then stored on-chip while the binding of intracellular proteins to the beads is monitored fluorescently. Based on this approach, the concentrations of specific intracellular proteins over five orders of magnitude (~50 pM to 1 μM) can be characterized [51].

Compared to conventional fluorescent flow cytometry, the droplet-based microfluidic flow cytometry does not need the steps of intracellular staining when the numbers of cytosolic proteins have to be measured. This advantage can address the issue of non-specific binding in the process of intracellular staining, which is definitely beneficial in protein quantification. However, in droplet based microfluidic flow cytometry, the dilution of targeted proteins occurs since the volume of droplets is roughly 0.1–1 nL, two or three orders larger than individual cells (~pL). Thus, protein-capture beads are required to enrich the concentration of targeted proteins, which, again, leads to the lack of effective calibration.

## 4. Large-Array Microwells (Microengraving)

**Figure 4:**
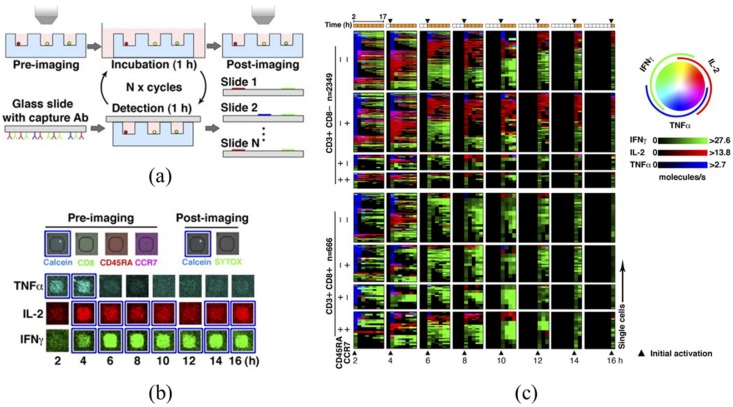
Large-array microwells (microengraving) enabling the quantification of secreted proteins of single cells. (**a**) Illustration of key steps in microengraving to monitor cytokine secretion in time; (**b**) representative micrographs of data evaluating cellular viability, phenotype, and secreted proteins; and (**c**) cytokine secretion kinetics of 3015 viable T cells. Each row reflects the dynamic activity of an individual T cell over time and the color wheel illustrates the type and relative magnitude of secreted cytokines. Reproduced with permission from [56].

Both conventional and microfluidic flow cytometry cannot evaluate the same single cells at multiple time points, which is of importance in immunology. To address this issue, Love *et al.* proposed the design of large-array micro wells (“microengraving”, 0.1–1 nL each) to isolate individual cells and quantify secreted proteins in a time sequence [53,54,55,56,57,76,77,78,79,80,81,82]. As shown in Figure 4, single cells, suspended in media, are deposited onto a large array (~20 × 50 μm^2^) of PDMS microwells. With the removal of excess cells, the microwells are then inverted onto a glass slide coated with a specific capture reagent. After an incubation period, the microwells are removed and applied to a second glass slide coated with a different capture reagent. The resulting microarrays are interrogated with laser-based fluorescence scanners.

After microengraving was initially proposed to quantify cellular protein expressions [76], several technical improvements were further realized, which are (1) the coating of specific macromolecules on the surface of PDMS wells to stimulate immune cells [54]; (2) the usage of the hybridization chain reaction to amplify signals resulting from sandwich immunoassays [55]; and (3) the retrieval of targeted single cells in specific micro wells [77].

Although microengraving can enable absolute quantification of multiple proteins secreted by individual immune cells, compared to flow cytometry, it requires the careful manipulation of glass slides without stimulating or even dislodging single cells within individual wells. In addition, it is also questionable that the micro environments formed within each microwell may be different from *in vivo* situations and, thus, the characterized protein levels cannot reflect the *in vivo* metabolisms of immune cells.

## 5. Large-Array Micro Chambers (Barcoding Microchips)

**Figure 5:**
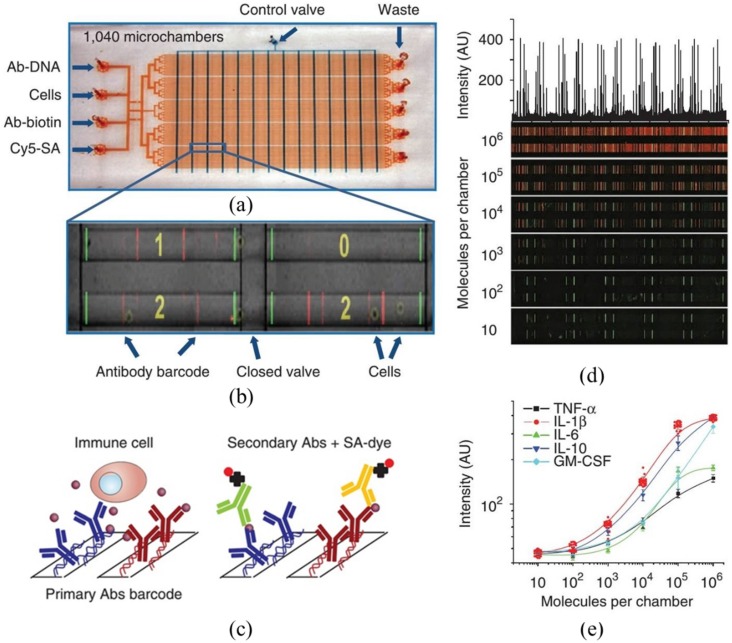
Large-array micro chambers (barcoding microchips) enabling the quantification of both cytosolic and surface proteins of single cells. (**a**) Image of the microfluidic device including flow channels (**red**) and the control channels (**blue**) with input and output ports labeled; (**b**) an image of cells trapped within individual chambers coated with antibody barcodes; (**c**) schematic of the barcode arrays enabling the detection of secreted proteins from single cells; (**d**) scanned fluorescent images used for the antibody barcode calibration measurements using spiked recombinant proteins; and (**e**) recombinant protein calibration curves for TNF-α, IL-1β, IL-6, IL-10, and GM-CSF. Reproduced with permission from [58].

Although the microengraving setup can enable absolute quantification of secreted proteins of single cells, this approach cannot be effectively used to measure cytosolic proteins. To address this issue, Heath *et al.* proposed large-array micro chambers (single-cell barcoding microchips), which can be used to assay both cytosolic and membrane proteins of single cells. As shown in Figure 5, the single-cell barcoding microchips consist of thousands of individually addressed micro chambers to conduct single-cell trapping, lysis, capture of targeted proteins by pre-printed antibodies on the surface of the chambers, enabling quantitative measurement using fluorescent immunosandwich assays [58,59,60,83,84,85,86,87,88,89,90,91,92,93,94,95] (see Figure 5).

The key feature of this technique is the preparation of spatially-encoded antibody barcodes enabling the device multiplicity. Since antibodies suffer from long-term storage issues, the barcodes were initially patterned as ssDNA barcodes. A cocktail of antibodies labeled with complementary DNA oligomers were then used to transfer the DNA barcodes into antibody barcodes, just prior to running experiments. This approach has been used to monitor multiple key cytokines of single cells, locating significant cellular heterogeneities in both immunology [58] and tumor biology [59,88].

This approach is the most powerful microfluidic system enabling the absolute quantification of both surface and cytosolic proteins of single cells in large arrays. However, there are also several practical concerns. First, this microfluidic system is a leaky system and when running experiments, large portions of single cells may stick to the bottom surfaces of cellular inlets. This limitation renders the quantification of cell types with limited numbers (e.g., circulating tumor cells) full of challenges. The second concern is the potential denature of targeted antibodies in the step of cellular lysis. In addition, to form individual chambers for single-cell analysis, it cannot be easily further scaled up to increase the throughput. Since this system requests complicated operation steps to form individual chambers for single-cell analysis, it cannot be directly scaled up to further increase the throughput.

## 6. Discussions and Future Development

In this study, we summarize key developments of microfluidic platforms enabling the quantification of single-cell proteins. Although significant improvements have been made within the last decade, there is still significant room for research since an ideal tool of single-cell protein quantification featured with absolute quantification, ultra-high sensitivity, and throughput is still not available.

Absolute quantification is a key requirement for single-cell protein assays. Without the capabilities of absolute quantification, the protein levels measured by different approaches cannot be effectively compared. For instance, microfluidic fluorescent flow cytometry can only provide semi-quantitative results by reporting levels of fluorescent intensities of flowing single cells. Although calibration beads have been widely used in the quantitative flow cytometry, they can only, to an extent, produce reliable results in quantifying membrane proteins, while we still lack effective calibration beads for absolute quantification of intracellular proteins in flow cytometry.

As to the issue of ultra-high sensitivity, large-array microfluidic devices suffer from the issue of sample dilutions since the reaction volumes for single cells are roughly 0.1–1 nL, two or three orders higher than the volumes of single cells (~1 pL). Further volume decrease in the trapping wells or chambers is not appropriate since it may lead to higher percentages of empty wells without cells and also affect the normal metabolisms of individual cells. To address this issue, fluorescent nanoparticles with higher fluorescent intensities may be used to replace conventional fluorescent probes. In addition, antibodies labeled with nuclear acids, which can be further amplified and quantified in digital PCR, can be further incorporated in the field of single-cell protein quantification.

The third issue is the throughput. Although the current microfluidic devices are treated as high-throughput approaches (e.g., ~1000 cells per second for flow cytometry), they are still not high enough. Taking the study of tumor heterogeneity as an example, individual cells within biopsy samples (~1 million cells) are preferred to be processed one by one. For the microfluidic flow cytometry, which inherently functions as a serial processor, further development should extend the capabilities to parallel processing. As for microfluidic large-array devices which are featured with parallel analysis of single cells, more work should focus on the device scale-up issue, definitely, requiring extensive optimization.
